# “If only balls could talk…”: barriers and opportunities to participation for students with blindness and visual impairment in specialized PE

**DOI:** 10.3389/fspor.2023.1286909

**Published:** 2023-12-15

**Authors:** Stefan Meier, Brigitta Höger, Martin Giese

**Affiliations:** ^1^ Centre for Sport Science and University Sports and Centre for Teacher Education, University of Vienna, Vienna, Austria; ^2^ Centre for Sport Science and University Sports, University of Vienna, Vienna, Austria; ^3^ Department of Inclusive Education, University of Education Upper Austria, Linz, Upper Austria, Austria; ^4^ Department of Natural and Human Sciences, Heidelberg University of Education, Heidelberg, Baden-Wurttemberg, Germany

**Keywords:** physical education, blindness and visual impairment, special schooling, barriers, participation and inclusion, awareness raising, adapted physical education

## Abstract

**Purpose:**

For children with blindness and visual impairment (BVI) of all ages, disability sport and/or regular Physical Activity (PA) are deemed beneficial, promoting physical and mental health as well as increasing wellbeing and life satisfaction. In this regard, Physical Education (PE) serves as a foundation to regular and lifelong participation in PA, mainstream and/or disability sport. Research points towards manifold participation barriers for children with BVI in PE, which so far have mainly been investigated in inclusive settings and from the perspectives of sighted parents, teachers and peers. Consequently, people with BVI frequently consider PE a missed opportunity for lifelong PA. As transitioning from general to special schooling deems the only alternative to continue their education, questions arise in how far and in which ways specialized schools manage to accommodate their needs in PE. To address these gaps in literature, we investigated BVI students' perceived opportunities and barriers to participation in PE within a specialized school setting and their imaginations for possible (digital) improvements and solutions.

**Materials and methods:**

Within the framework of Inclusive and Youth Participatory Action Research, we adopted the Mosaic Approach to investigate a sample of 19 students aged 14–20 at lower and upper secondary level in a specialized school in Austria. Data material included audio-recordings of interviews, student-guided school tours, photographs of significant places and objects and field protocols. The analysis was conducted with Interpretative Phenomenological Analysis.

**Results and conclusion:**

Through the analysis, we identified three themes. The data material firstly revealed the complex intricacies of how PE teachers can act as facilitators and gatekeepers to autonomous PA. Secondly, material norms function not only as barriers to participation even in a specialized school setting, but also constitute the basis for social hierarchies between students with various degrees of visual impairment. Thirdly, students imagined manifold digital solutions to enhance participation derived from their perceived barriers. The findings contribute to amplifying BVI individuals' voices and provide revealing insights in how participation in PA is enabled and prohibited for students with BVI which can not only help to improve specialized but also inclusive settings.

## Introduction

The global inclusion movement as well as the UN Convention on the Rights of Persons with Disabilities (1) lead to an increasing awareness for the need to overcome the exclusion of marginalized and discriminated groups in society. In that respect, people with disabilities in sport are increasingly becoming the focus of social and scientific considerations. Both mainstream sport (2) and disability sport (3) are commonly acknowledged for their high potential to assume social responsibility for the development of an equitable society (4), even though empirical evidence that such can be achieved has been lacking to date (5).

At the same time disability is conventionally underexplored in sports science research, both in competitive and in elite sports for people with disabilities. Disability sport primarily comes into focus where it submits to the immanent performance and enhancement logic of sport that can be exploited by the media, whereas risks of disempowerment usually go unnoticed (6, 7), as does the critical discussion of ableist implications (8). However, for people with blindness and visual impairment (BVI) of all ages disability sport and/or regular Physical Activity (PA) are deemed beneficial, promoting their physical and mental health as well as increasing wellbeing and life satisfaction (9). Moreover, regular PA can potentially improve BVI people's spatial orientation skills (10) and their sense of hearing (11). In contrast, international research repeatedly shows that people with disabilities generally show lower levels of PA than people without disabilities (12). Specifically, people with BVI display lower levels of motor competence (13, 14). Children and youth with BVI are less active in (disability) sports and regular PA and suffer from obesity more frequently (15, 16) compared to their sighted peers. Since low levels of motor skills can have a negative impact on participation in sports and movement culture, children with BVI need as much motor skills development as possible, including in the school subject Physical Education (PE). However, in terms of participation, children with BVI face particular barriers, i.e., lack of specialized sport activities and/or specialized instructors, the fear of getting injured (17, 18). In terms of regular PA, research suggests that PA behaviors develop continuously from childhood to adulthood (19). Hence, it is important to seek an understanding of youth PA behaviors to improve PA among individuals with BVI in adulthood. For youth with BVI, the most likely environment to learn about and participate in PA is school-based PE (20). Thus, PE can be seen as a unique possibility to serve a foundation to regular (and lifelong) participation in PA, mainstream and/or disability sport, and thereby can help to make a positive contribution to social, mental and physical well-being (21). However, students with disabilities continue to encounter extensive barriers both in inclusive and segregative schooling. Truly inclusive experiences are often inaccessible to these students (22).

In terms of research methodology, it should be noted that such research is typically conducted *on* students with disabilities (23, 24) and tends to emphasize the perspectives of nondisabled peers, parents, teachers, and experts, while systematically ignoring the voices of students with disabilities themselves (25). This is problematic as it limits our understanding of these students' thoughts, feelings, and experiences, which are central to designing mindful settings that allow for participation (26).

With regard to education, for children with BVI PE can serve as an important facilitator of physical and mental health as well as life satisfaction (9, 27). Yet, research points towards manifold participation barriers for children with BVI in PE. However, these barriers and possible solutions have so far mainly been investigated from the perspectives of sighted parents, teachers and fellow students (23, 28). In fact, while such research efforts have accumulated vast insights into how able-bodied individuals perceive and conceptualize BVI in PE and sports in general, the experiences and perspectives of individuals with BVI themselves have been conspicuous by their absence. This is problematic, as it limits our understanding of “inclusive experiences” and feelings of children and youth with BVI, which should be at the core of interpretations of inclusivity in general (26, 29). Consequently, PE has remained a context in which many students with BVI do not experience feelings of belonging, acceptance or value and consider it a missed opportunity to initiate lifelong PA (30–32).

Hence, to amplify their voices, research needs to further uncover BVI students' experiences of participation in PE, sports and PA from their very own perspective. In that respect, Giese (33) investigated BVI students' subjective constructions of participation barriers in inclusive PE. Since transitioning from general to special schooling deems the only plausible decision for many students with BVI to continue their education (34), questions arise in how far and in which ways specialized schools manage to accommodate the needs of students with BVI in PE. Furthermore, all studies to date have been conducted in removed interview settings whereas “none of that research explores disabled students' intersubjective experiences of belonging, acceptance, and value in the spaces where they find themselves” (35).

Against this background, we investigate the perceived barriers and opportunities to participation for students with BVI in specialized PE and how students with BVI imagine possible improvements and solutions in a participatory research approach. The results may help to improve opportunities of participation in inclusive PE settings as well as shed light on the potential shortcomings of PE in specialized settings in order to further strengthen the quality of education for students with BVI (1). In a wider context, the gained insights can potentially help to promote participation in sporting activities and thus increase opportunities for PA among children and youth with BVI, which will contribute positively to their health and wellbeing (1, 21). Through employing a participatory research approach, our study honors the UNCRPD's claim “Nothing about us without us”, fosters awareness for the concerns of people with disabilities (UNCRPD, Art. 8) and contributes to increasing social justice (36, 37).

## Materials and methods

### Methodological framework: participatory action research with students with disabilities

This research was conducted in the tradition of Participatory Action Research (PAR), particularly leaning on principles of Inclusive Participatory Action Research (IPAR) (36, 38) and Youth Participatory Action Research (YPAR) (37).

Following a social constructionist perspective, we understand disability as a socially constructed identity category similar to gender or race. We understand the existence and subjective experience of one's abilities as a fundamental facet of the human relationship to the world, in which individuals relate to their surroundings in an efficacious, deliberate and enjoying manner (39). In that respect, ableism describes the underlying system of beliefs, processes and practices of preferring assumed species-typical normative abilities over others, resulting in the discrimination of those who are deemed “less able” and/or “impaired” due to failing to fulfill said norm (40). As all participatory methodologies, IPAR aspires to be “emancipatory, empowering and democratic and to illuminate social problems” (36) and aims to reveal the individual experiences of people with disabilities in order to comprehend and emphasize their concerns and needs (36). Similarly, YPAR is founded on the everyday experiences of young people and follows the premise of embracing their potential by working *with* them in solidarity instead of *for* them to make the world “a more just, equitable, and humane place to inhabit” (37).

Our research presented in this paper is part of a larger project funded by the Austrian Ministry of Education, Science and Research (BMBWF). The funding pool supports Participatory Action Research projects in which students of all school levels and other potential actors in the field are actively involved in the research process and thereby contribute to research which would otherwise not be possible (41).

The overarching research project aims at the participatory development of digital assistive technology for students with BVI in PE and sports. In the project, students with BVI from a general lower and upper secondary school specializing in blindness and visual impairment, sighted students from vocational schools specializing in mechatronics and computer science as well as their sighted teachers join a team scientists from sports pedagogy and biomechanics and become co-researchers in a participatory research process (37, 38). In the spirit of (I/Y)PAR and the UN-CRPD's claim “Nothing about us without us”, students collaborate under the guidance of the scientists and develop digital assistive technologies for students with BVI in PE based on the BVI students' identified requirements and ideas (36). Developed prototypes are tested and refined jointly by sighted and students with BVI in PE, eventually will be presented to a wider audience and possibly serve as a starting point to making these assistive technologies available on a larger scale in the tradition of “open science” (42). The collaboration raises awareness for the life realities of individuals with BVI in the contexts of school and sports among sighted students and their teachers (1) and furthermore creates opportunities for empowerment and participation in the process of fostering inclusion and improving the situation of students with disabilities in PE and sports in the pursuit to increasing social justice in the educational system (1, 36, 37).

In this article we focus on the very first step of the project which aims to identify the perceived barriers and opportunities to participation of students with BVI in PE as well as their identified needs, requirements and ideas for improvement in a specialized school setting through—among other measures—digital assistive technology.

Our research questions are the following:

• How do students with BVI perceive barriers and opportunities to participation in PE in a specialized school setting?
• How do students with BVI imagine accessible PE in the (digitized) future?

### Sample

The respective school had been selected due to its longstanding expertise in teaching students with BVI and its openness to enter three-year-long extensive collaboration in the overall project. Participants for this initial investigation were purposefully recruited based on the following criteria: (1) being at least 14 years of age^1^, (2) attending the respective school specializing in BVI and consequently (3) being blind or visually impaired, (4) being willing to participate in an audio-recorded group interview and school tour. Students were invited to join the investigation with the help of their PE teachers and recruited by the researchers based on their interest to participate. The final sample consisted of *N = *19 students (12f, 7m) aged 14–20. 16 of them were visually impaired and three were fully blind at the time of data collection (Table 1).

**Table 1:** Characteristics of the students.

Pseudonym	Age	Gender	Degree of VI
Michael	16 years	Male	Visually impaired
Vanja	18 years	Female	Blind
Lina	14 years	Female	Visually impaired
Samira	16 years	Female	Visually impaired
Laura	16 years	Female	Blind
Emma	15 years	Female	Visually impaired
Ayse	19 years	Female	Visually impaired
Sarah	16 years	Female	Visually impaired
Zahra	14 years	Female	Visually impaired
Nuri	14 years	Female	Visually impaired
Kerstin	14 years	Female	Visually impaired
Luca	15 years	Male	Visually impaired
Emir	16 years	Male	Visually impaired
Noah	16 years	Male	Visually impaired
Maximilian	20 years	Male	Visually impaired
Liam	17 years	Male	Visually impaired
Elena	15 years	Female	Blind
Sasha	14 years	Female	Visually impaired
Nikita	14 years	Male	Visually impaired

We decided not to ask the participants for their medical diagnoses, but instead chose to ask them “How come that you are attending this school?” and “What is your vision like at the moment?”, which elicited an answer related to their vision in a broader social context. Some students gave their diagnosis, but others answered something along the lines of “Actually, I can see quite well…” followed by a description of their vision. Most interestingly, one girl described that according to her doctor she should be entirely blind, but in fact, she can recognize shapes and nobody knows how and why this is possible. It seemed to us as an ability quite important to her. Even though we acknowledge medical diagnoses do have a time, place and purpose, in our understanding, these descriptions were not only much more specific and informative than their diagnoses (which not all students were able to name), they were also remarkably revealing regarding the subjective meanings' participants assigned to their respective visual abilities.

All blind students had visited the respective school since primary level. All visually impaired students had received schooling in an inclusive setting during primary level and had transferred to the respective specialized school for BVI either at the transition to lower secondary level or during lower secondary level following recommendations of their teachers and due to self-reportedly not having their educational needs met in inclusive settings. 17 students had previously visited schools in Austria, one boy had transitioned from an inclusive lower secondary school in Germany and one girl had attended an inclusive primary school in Syria and inclusive lower secondary school in Austria before transitioning to the specialized school. Written informed consent was obtained from all students before the interviews. Data acquisition took place at the school during PE lessons or recess. No teachers were present for the entirety of the interviews.

### Methods of data collection

Neither YPAR nor IPAR prescribes the utilization of specific research methods. Instead, methods of data acquisition and analysis have to be selected as appropriate to the specific research context. Since PAR methodologies have originated from various contexts of human rights activism advocating for social change and the liberation of marginalized groups, the critical reflection of power structures forms an integral part of designing and conducting any PAR project (43). In our case, we carefully considered how intersecting power structures of dis-/ability, age and formal education could affect the course of the project and data acquisition of the first project phase in particular.

Under these presuppositions, we found Clark's Mosaic Approach (44, 45) to be most suitable for our research endeavor. Originally developed in the context of researching young children's life-worlds in pedagogic institutions, the Mosaic Approach is a multi-method, participatory, reflexive, practice-oriented and adaptable approach which focuses on the lived experiences of participants and considers children and youth to be “competent, active meaning makers and explorers of their environment” (44). Following Burke (46), the Mosaic Approach proposes that “rather than being viewed as a neutral or passive ‘container’, if recognized at all, the school building, its various rooms and spaces, the walls, windows, doors and furniture together with outdoor ‘nooks and crannies’, gardens and open spaces are considered here to be active in shaping the experience of school and the understanding of education”. This understanding corresponds to Haegele and Maher's conceptualization of educational spaces in the context of inclusion and BVI (35): “For us, education spaces are not fixed or absolute. Material spaces, such as schools, classrooms, playgrounds, and gymnasia, are not containers of human activity or blank canvases. Rather, they are socially constructed and (re)produced through human interactions”. Being at school, as much as the learning that takes place in them, must be considered an embodied experience to be reconstructed as the relationship between spaces, people and objects (45).

Specific methods of data collection were carefully selected as a result of critical reflections upon which kinds of methods would actively involve the students, yet were equally accessible to all students from the sample. For our study this meant avoiding methods that rely solely on students' visual abilities while simultaneously providing a variety of ways for them to explore their ideas and engage in the conversation about their experiences in PE as well as using age-appropriate language in every step of the research endeavor (38, 47, 48). As a result of these reflections, we decided to conduct guided group interviews followed by student-led school tours as well as collect photographs of places and objects pointed out as relevant by the students and field notes.

Group interviews were performed in an empty classroom/gym hall between one researcher (first or second author) and groups of two to three students following an interview guideline based on Clark's (45) dimensions of people, places and objects comprising students' embodied experiences of schools, which we applied to the context of PE. After eliciting a broader description of their PE lessons, students were asked to elaborate on the persons involved in PE and their respective role (e.g., fellow students, teachers, assistants, etc.), describe the places and objects that were frequently used in PE (e.g., gym, garden, sports court, gym equipment) and recollect lessons they particularly liked and disliked as well as imagine a perfect PE lesson. We purposefully did not address any impairment-related adaptations or perceived barriers in PE directly and only at the very end asked them how it came about that they visited this particular school, to describe their eyesight and how they particularly perceived it during PE. This was done for two reasons: Firstly, we wanted to avoid reproducing a power dynamic in which students were stereotypically labeled as disabled by non-disabled, adult professionals, which might have left them feeling disempowered and objectified (49). Secondly, as a precaution to avoid the reproduction of ableist notions and inadequate, unjustified assumptions of normality (50), we did not want to unnecessarily problematize BVI in ways that might not necessarily be relevant to the students themselves (37). In that way, students had the opportunity to give a more authentic account of how they perceived their life reality as BVI individuals in the context of PE and sports.

After the guided group interview, students were prompted to take the researcher on a school tour and show them the spaces in which PE lessons usually take place (47). Students were asked to describe each space in their own words, point out what they liked or disliked about it and why, and describe objects that were of particular importance to them. During these school tours, the researcher took images of spaces and objects of particular importance to the students^2^. The end of the school tour was marked by an imaginary future scenario in accordance with Clark (44) (“Imagine you could time-travel to 2050…”), asking students how they imagined the future of PE and which places to keep, expand, change and add. Data were complemented by reflexive field notes written by the researchers.

### Data handling and analysis

Group interviews and school tours were audio-recorded, transcribed verbatim, grouped with respective photographs and field notes and imported into the qualitative data analysis software MAXQDA (51). Students' names were replaced by pseudonyms to ensure anonymity. Subsequently, we utilized Interpretative Phenomenological Analysis (IPA) (52) to analyze the data. IPA explores the embodied experiences of individuals and how individuals assign meaning to their personal and social environment, making it highly compatible with the methodological foundations of the Mosaic Approach (44, 45, 47) and a suitable method to answer our research questions.

IPA incorporates phenomenological (i.e., centering on individuals' lived experiences as personal accounts rather than objective descriptions), hermeneutic (i.e., relying on the researcher's interpretation for gaining insight in said experiences) and idiographic (i.e., emphasizing on each individual's experience through intensive analysis) elements. In other words, IPA is a method to gain understanding of the individual lived experiences of participants through the process of the researcher interpreting individuals' meaning making processes based on their individual accounts. The analysis was conducted in several steps as recommended by Smith et al. (52). Firstly, we (re-)immersed ourselves in the data material through multiple rounds of listening to the audios and reading the transcripts to familiarize ourselves with the data while secondly, adding comments and highlighting potentially significant passages. As a third step, the first and second author reduced the data for each case (data from one group of students including researchers' comments) to emergent themes, reflecting participants' statements as well as researchers' interpretations. Lastly, we identified convergent and divergent themes through comparison between cases. Recurring themes were then discussed among all three authors in terms of traceability, while divergent themes were debated until consensus was reached.

### Quality of data

We applied several strategies to assess the quality of data (52, 53). Sensitivity of context was ensured by a rigorous and theoretically informed reflection of power dynamics in research design and researcher positionality. Moreover, results were presented including a considerable amount of insightful quotes from the data. Commitment and rigor were established by deriving the methods of data acquisition as well as the detailed construction of the interview guide from the methodological framework of the study as well as the authors' commitment to enter a three-year-long research collaboration with the participants' school. Transparency was addressed by the detailed description of recruitment, data collection and analysis whereas coherence was established through the congruence between theoretical foundations, research questions and methodological considerations.

Lastly, the potential impact and importance of the study lies in its capability to not only provide further insight into an existing issue but to also contribute to possible solutions in a way that empowers participants along the way, but will eventually be up for judgment by the scientific community and after finalizing the project (53).

## Results

The analysis of the data material revealed three themes regarding how students with BVI perceive barriers and opportunities of participation in PE in a specialized school setting. In accordance with the theoretical and methodological background, what turned out to be perceived as a barrier or opportunity to participation is constituted through intertwined constellations between spaces, objects and people (45).

### “She said, she would think about it…”—participation and autonomy

The first theme emerging from the analysis were the ways in which students negotiated questions of participation in terms of the ways they were granted autonomy when moving through the various spaces of the school, particularly those associated with PE. Through the interviews we learned that the school tried to increase students' autonomy by designing the school as a barrier-free, secure space for students with BVI and by providing extensive mobility training at the school grounds, so that particularly blind students would be able to navigate through the school spaces without assistance.

Lina: “[…] the sports hall, the gym or outside is a safe space for the students, even the blind, they know the facilities well, they have had mobility training there or they know where to go and whom to ask, if they need assistance. And then, we or the teacher help those blind or more visually impaired students […]” (Interview 2, 56-56)

With particular regard to PE-related spaces and opportunities for PA, the school had established several spaces for students to engage in PA, such as outdoor playgrounds, sports courts and a small school gym, a room with fitness equipment such as cardio machines and weights. PE teachers also had established a “fitness certificate”, an authorization which students had to acquire in order to use the school gym independently. Teachers made use of the school gym during PE lessons, but the certificate allowed students to access it without a teacher present during recess or in their spare time^3^. For the certificate, PE teachers would provide students with specific training which involved basic orientation to move around the school gym as well as more specialized knowledge on how to use the machines in an appropriate and safe manner. In that sense, PE teachers play a major role in empowering students and facilitating autonomous participation in PA within the context of school, but also provide opportunities for students to prepare to use public gyms outside of school independently. However, as it turns out PE teachers could also easily become gatekeepers to PA, in case certain propositions are not being fulfilled, leading to feelings of frustration in the students. Upon further investigation and although PE teachers may have had plausible reasons, it remained unclear to the students why the proposition of the fitness certificate was not unfulfilled by their teacher. As one student describes:

Vanja: “[…] She [the PE teacher] told us in the first couple of lessons, that if we are not happy with something, we should tell her and make suggestions to change it. So, we asked, if we could get our fitness certificate, so we could go to the fitness room any time we want without a teacher. She said, she would think about it. But up until now, nothing has happened.” (Interview 1, 23-23)

Students described how their request to acquire their fitness certificate was not met, despite of their teacher asking them to share their wishes for PE and voice their opinions by making suggestions. One student points out:

Luca: “Personally, I’d like to see us discussing our wishes and needs in PE, and seeing them being recognized as relevant.” (Interview 5, 99-99)

Hence, the particular example of the school gym illustrates how PE teachers can simultaneously adopt the role of facilitators as well as gatekeepers to autonomous PA for students with BVI in a specialized school setting. Especially if students are left in the dark about reasons for denying them access, it may perpetuate feelings of exclusion and powerlessness. Furthermore, students described that the machines at the school gym were associated with certain barriers themselves:

Michael: “[…] Maybe there could be like microchips in the training machines.

Researcher: What would you use those for?

A: Like for reading off the screens, it would be important for blind people to have like sound or a speech output. I don't know.” (Interview 1, 94-94)

Students described that particularly digital cardio-machines were designed with sighted users in mind, as their operation required reading from a screen. As a consequence, in order for the students to use these machines to their full extent, they were yet again dependent on the assistance of their sighted PE teachers or partially sighted peers. Hence, when it comes to engaging with and participating in the movement activities of school spaces, the school gym serves as an illustrative example for how the characteristics of stakeholders' actions and properties of spaces and objects constitute opportunities and barriers to autonomous participation in PA. Students strongly expressed the desire for their perspectives to be taken seriously to be self-determined agents within PE/PA. In light of the above-mentioned intricacies, notions of facilitating and prohibiting PA for students with BVI lie closely together and attempts to foster autonomous participation can easily become lost opportunities.

### “Sometimes the floor is louder than the ball…”—participation and material norms

The second theme centered around the question of how particular material norms in spaces for PA played a part in constituting opportunities and barriers to participation in PE and PA. In Austria, the so-called OENORM (54) is a collection of legally binding documents containing technical norms and standards by the Austrian Standards Institute. They determine specific requirements, procedures, measurements and guidelines for a variety of areas, among others public and school sports facilities, in order to ensure their security, quality and compatibility. Our analysis brought to light that the material conditions such as space, noise, lights, colors, etc. as well as their interplay, which are largely determined by these legally binding norms, are of crucial importance for participation in PE.

Although the OENORM (54) aspires to guarantee accessibility to (school) sports facilities by ensuring appropriate lighting, colors of floor markings, etc., the students criticized exactly these norms that should ensure their participation.

For instance, one student mentioned:

Noah: “Uhm what else… the floor should have better color distinction. The lines should be thicker. And the playing field should be brighter and the edges should be darker, maybe black, so you can recognize things better. I mean it is already dark, but not really dark.” (Interview 5, 230-230)

While this particular student expressed his requirement for better color distinctions of the playing field, he and his peers also pointed out throughout the interviews that visual perception can be very different between students, deducing that a visual “one size fits all” kind of solution may be inherently problematic. As a consequence, students imagined several digital and analogous solutions to foster participation when it comes to recognizing the lines of a playing field, which were not reliant on eyesight, for instance haptic stimuli:

Maximilian: “[…] Maybe there could be a small notch where the line is, or something else that makes it easier to feel the line. It may be difficult to feel it through the shoes though […]” (Interview 6, 36-36)

Another example for problematic material norms was described with regards to the gym floor. It turned out that certain noises coming from the gym floor itself were perceived as a hindrance to their participation in certain games. One student mentioned:

Luca: “[…] The floor creaks really loudly. When you walk, you can hear that it’s quite old.

Researcher: And is that a problem for you or is it just an unpleasant sound?

L: It bothers me when we play soccer, because sometimes the floor is louder than the ball.” (Interview 5, 172-172)

Students also mentioned that standardized diving objects made it more difficult to participate in diving activities depending on their visual abilities:

Laura: “[…] It takes a bit longer. You have to dive all the way down to the ground and as a blind person, you have to like frisk the entire floor and it takes quite long with your breathing. It’s made for sighted people. I mean blind people can do it too, but it’s a bit more difficult.” (Interview 2, 136–141)

Thus, the analysis uncovers that the inherent material norms of sport areas not only fail to consider the prerequisites of students with BVI for participation in PE and thus constitute a barrier. It further becomes evident that the orientation towards these norms itself perpetuates the ableist distinction between sighted, “more” or “less” visually impaired and fully blind students and creates the basis for blind students being in need of assistance in the first place. Hence, even in a specialized school material norms seem to pre-determine which abilities are required in order to participate.

### “If only balls could talk…”—Imagining participation in digitized futures

The third theme centered around the students' imagined futures for PE. Throughout the interviews students described in great detail which aspects of PE were not accessible for them how and why, and which aspects they found bothersome in light of their impairment, despite the specialized setting they found themselves in. In the last section of the school tour students were encouraged to imagine the future of PE (44), regardless of how unrealistic their ideas may sound. Throughout the analysis it became evident that their imaginations of analogous and digital innovations were strongly informed by and directly correlated to the barriers they identified during the interviews.

One of the most central topics in PE lessons turned out to be ball games. Students described how sound balls (i.e., balls with a small mechanical bell inside of them which make a ringing sound when in motion) were frequently used in various kinds of ball games such as soccer, basketball or goal ball.

Nuri: “We have this ball that makes a ringing sound.”

Kerstin: “There is a little bell inside and for example, if you roll or kick it, it makes a noise.”

Nuri: “But when the ball stops, blind students don’t know where it is and cannot get it back. If only balls could talk… (laughs).” (Interview 4, 53–55)

Whereas sound balls were on the one hand deemed as a possible solution to make ball games (more) accessible to students with BVI, the solution was only partial and came with further potential challenges. Besides not being able to locate the ball whenever it came to rest, the sound was frequently reported as generally too soft or too brief. As a result, one blind student even described how she was offended by her teacher for not performing as expected when she could not hear the ball's sound:

Michael: “It was quite noisy all around me. I could not concentrate well and could not hear where the ball was. Then the teacher said in front of everybody that I had issues with orientation, because I did not know where the ball was. She said I did not hear well.”

Vanja: “Well, when everyone around you is screaming, you just can’t hear the ball ringing. Of course you don’t have any sense of orientation.” (Interview 1, 117–118)

As a solution, students imagined sound balls in different sizes, more vibrant colors and various consistencies that had the capacity to “talk”. With the capacity to “talk”, students referred to sound mechanisms that were more adaptive to specific scenarios and game contexts regarding the timing, duration and volume of sounds. Furthermore, sound balls should have a higher degree of sensitivity when responding to motion patterns.

Similarly, students reported current strategies to make soccer goals locatable through sound, but wished for basketball hoops and soccer goals that could “talk” as well, meaning that they are locatable through sounds and audibly responsive to the game.

Luca: “Basketball hoops should make sounds, maybe soccer goals too. I mean, usually, the goalie knocks at the frame to indicate left and right.” (Interview 5, 230)

Nuri: “The hoops should have lights that turn green when you hit and red when you miss. Or they should talk.”

Kerstin: “Yeah, that would be cool!”

Luca: “Yeah, for blind people that would be cool!” (Interview 4, 139–141)

Another predominant area for possible digital assistive technology were activities such as running, biking, rollerblading and ice skating. In that context, an aspect that became evident through the analysis was the question of interpersonal relationships and their significance for participation. Well-meaning teachers were reported to make an effort to enable blind students to participate in running activities through lesson arrangements in which blind students were forced to depend on the assistance of their sighted teachers. While acknowledging their need for assistance, such practices however were perceived as highly segregative and exclusionary and thus hindering PA as well as social participation. Blind students pointed out that for them the essence of participation lies in being with and possibly being assisted by their (partially sighted) peers instead of having to depend on their sighted teachers.

Samira: “Yeah, I think that blind students should not always have to be stuck with the teacher, for instance when we go for a run outside. Blind students should be with the other [visually impaired] students and be part of the group.”

Laura: “Yes, because when a blind student runs by themselves, they can easily miss an obstacle and bump into something. So it’s better to always have a [sighted] partner with you.” (Interview 2, 46–47)

Based on the perception of this barrier, students mentioned very specific pieces of technology that would foster participation while engaging in PA. They imagined wristbands with sensors that can detect obstacles and provide acoustic or sensory feedback.

Lina: “They should make something for obstacle detection for running. Like when blind people go running by themselves so that they don’t bump into things.”

Samira: “Yeah, like a wristband.”

Lina: “…that beeps when you run and come too close to a tree for example.” (Interview 2, 310–312)

In that sense, while students reported assistance from a sighted partner to be a feasible option to enable blind students to participate in such activities, their suggestion for a digital solution still attests for their desire for a higher degree of autonomous participation.

## Discussion

This study investigated BVI students' perceived barriers and opportunities to participation in PE within a specialized school setting and their imagined (digital) improvements and solutions. Imagined improvements and solutions were directly derived from barriers to participation, which points towards the fact that even though PE teachers make efforts to foster participation in sporting activities, students still see room for improvement in order to accommodate their requirements and wishes for inclusion.

Even in the investigated specialized school setting, students with BVI reported barriers to participation in PE, which closely corresponds to reports from students with BVI in inclusive settings (26, 33, 55). Although the students in our study reported that their opportunities for participation in PE drastically improved after transferring to the specialized school, the reported barriers still caused the individuals in this study to feel frustrated and their feelings and needs disregarded, as reported in previous studies (28, 30, 56, 57). Hence, establishing so-called “specialized” settings in which students are encouraged to voice their needs and concerns does neither guarantee comprehensive accessibility and participation nor unclouded feelings of inclusion, self- or co-determination (31). As a result, PE can still easily deteriorate into a missed opportunity for individuals with BVI to increase long-lasting appreciation for PA (32) and may fail to initiate participation in mainstream and/or disability sport and to foster physical and mental health as well as wellbeing and life satisfaction (9, 21, 27).

The analysis showed that PE teachers play a crucial role in the process of fostering participation in specialized settings, similarly to inclusive settings (30). While PE teachers in this study were perceived as invested facilitators who are willing to accommodate students' perspectives and wishes, they simultaneously could easily act as gatekeepers to PA. Even in the specialized school setting under investigation, students with BVI were only granted access to sports-related spaces under specific circumstances (i.e., the fitness certificate), and if so, identified barriers that prohibited them to fully engage in PA (i.e., cardio machines with screens). Thus, even in specialized settings students with BVI may not be able to fully participate in PA within the bodies they inhabit, as has previously been reported by Titchkosky (58) for inclusive PE settings and spaces. As a result, bodies deemed as “disabled” are essentially constructed as unable to occupy sports-related spaces. Such findings must be considered as problematic, as they raise questions as to whether creating specialized PE settings is solely a “lip service […] being paid to the notion [of inclusion] at the level educational […] practice (59). Reported invitations of the PE teachers to “make suggestions for improvement” may deteriorate into empty promises and result in PE teachers repeatedly being placed at the center of students’ engagement and enjoyment of PE (20, 31). These findings confirm that even if intentions may be good, “unintended and often unnoticed consequences associated with integrating students with visual impairments into poorly accommodated activities can have detrimental effects” (30).

Moreover, material norms regarding the construction and design of spaces for PE/PA strongly contribute to excluding students with BVI from fully participating in sporting activities. These norms implicitly corroborate assumptions of physical normality and normal abilities and thus can easily perpetuate a deficit-oriented perspective, resulting in the discrimination of those who are deemed “less able” due to failing to fulfill a certain norm (40). Surprisingly, the analysis confirmed that these norms are in place in inclusive as well as specialized settings. Thereby, ableist notions of physical normality and assumptions of normal abilities even trickle into specialized settings, which have been established to accommodate the requirements of their target group. Less surprisingly, the students reported numerous barriers which specifically resulted from the uncritical application of those material norms to “specialized” PE spaces. As a result, the declaration of creating sports-related spaces as “safe” for students with BVI is in need of critical reflection (60).

BVI students' feelings of inclusion and belonging as well as their opportunities of participation stand in direct contradiction to the perpetuated universal notion of “one size fits all” when it comes to constructing and designing sports-related spaces according to material norms. The uncritical and ongoing adoption of material norms perpetuates ableist social hierarchies and reinforces a number of exclusionary dynamics, as reported in previous studies (26, 57). Partially sighted students value and appreciate their own visual abilities regardless of any diagnosed impairments, as should their abilities be valued by others, e.g., in teaching and learning in PE. These students wish for adaptations that enable them to make best use of their vision and want to fully rely on their visual abilities when accomplishing given tasks. Dismissing their visual abilities and treating them as “essentially” blind may lead to feelings of disempowerment and frustration. In other words, they express the wish to relate to their surroundings in an efficacious, deliberate and enjoying manner (39).

On the other hand, continuously creating environments in PE that require a certain degree of vision in order to accomplish tasks contribute to socially discriminating against blind students as they become reliant on the assistance of either their sighted teachers or partially sighted peers. Being forced to constantly rely on the assistance of others perpetuates a deficit-oriented perspective on respective individuals, resulting in the discrimination of those who are deemed “less able” due to failing to fulfill a certain norm (40). Students reported that having to rely on the teacher reinforces feelings of social exclusion from their classmates for blind students. At the same time, continuously providing assistance for blind students may have negative ramifications for partially sighted students such as feelings of obligation or (more implicitly) feelings of separation (61). Ultimately, the uncritical adoption of material norms perpetuates social ability-related hierarchies creating tops and bottoms, which marks a major characteristic of ableist orders as individuals are ranked in relation to their performances of abilities (62).

Lastly, students with BVI turned out not only being able to clearly identify opportunities and barriers of participation in PE as well as express their perspectives and wishes, they were also immensely resourceful regarding specific digital and analogous innovations that could be of assistance when participating autonomously in PE/PA.

### Limitations and strengths

The presented study shows specific strengths and limitations, which will be critically reflected upon in the following. A particular strength of the study is that it addresses the proposed research gap in a methodologically innovative way. By adopting a participatory approach, our study takes into account the inherent power dynamics that come into play when researching in the field of sports, disability and adolescence. By being situated within a qualitative research paradigm, our study provides the individuals under investigation—in our case students with BVI—with the opportunity to express experiences, feelings and opinions from their very own perspective (63). Furthermore, by conducting group interviews and guided school tours to explore the PE-related spaces in a specialized setting, our study addresses an attested shortcoming of previous studies by conducting our data collection in the very spaces that we are investigating (35).

On the other hand, our study shows certain limitations. Firstly, due to the design of the entire research project the investigation took place in only one specialized school. Consequently, the transferability of the findings may be limited due to the specificity of our sample. Thus, further research in other schools specializing in BVI will be needed. Secondly, due to the qualitative nature of our study, the experiences of these participants may not be representative for PE experiences of students with BVI in other contexts. Typically, qualitative inquiries, including (I/Y)PAR studies, investigate samples in order to provide sufficient cases for the development of meaningful points of similarity and difference between participants, but not to get overwhelmed by the amount of data generated (52). Thirdly, to further remove BVI students' barriers to participation in PE it seems reasonable to investigate both the students and the teachers' perspective (64), which is one of the following steps of our study. Lastly, we approached the examination of BVI students’ perspectives towards participation in PE by using our considerable previous knowledge in sports pedagogy. This positionality should be critically considered when consuming this research, as BVI students' perceived barriers and opportunities to participation in PE and how they imagine possible improvements and solutions has been investigated through these filters.

## Conclusions and future directions

The purpose of this study was to enhance our understanding of BVI students' perceived barriers and opportunities to participation in PE within a specialized school setting as well as their ways of imagining (digital) improvements and solutions. The unique and valuable contribution of the study is how students with BVI imagined possible improvements and solutions in this regard. The analysis of the data material revealed three themes, which identify barriers, opportunities, and imagined improvements and solutions that were viewed as critical towards PE participation in a specialized school setting from BVI students' point of view. The findings suggest that participation in PE would benefit from acknowledging students' voice, so they can interact fully with the PE spaces and the activities within them. In this regard, students' autonomy, a critical reflection of ableist notions intertwined in spaces, objects and stakeholders, and a critical examination of students' suggestions towards barriers are of critical importance. These findings provide support for the assertion that research should honor the UNCRPD's claim “Nothing about us without us” as it will amplify students’ voices and will foster awareness for the concerns of people with disabilities (UNCRPD, Art. 8). Therefore, future (participatory) research should rely on students' lived experiences as potential signposts when it comes to removing barriers to participation. Teachers and their students should deliberately collaborate to co-construct supportive (64), accommodating environments that allow for social and pedagogical inclusion in specialized as well as inclusive PE settings. This may generate learnings for inclusive PE settings as well. In a wider context, such insights could not only help to promote participation in sporting activities and thus increase opportunities for PA among children and youth with BVI, but may also be crucial in contributing positively to their health, wellbeing and life satisfaction long-term and on a larger scale (1, 21).

## Data Availability

The raw data supporting the conclusions of this article will be made available by the authors, without undue reservation.

## Footnotes

^1^In Austria, 14 marks the age at which adolescents are legally competent to give informed consent to participate in research studies without the approval of a legal guardian.

^2^For reasons of data protection, the students themselves were not in the images.

^3^As most specialized schools in German speaking countries, the school has a boarding home for students living further away.
